# Enhanced anti-cancer effects of oestrogen and progesterone co-therapy against colorectal cancer in males

**DOI:** 10.3389/fendo.2022.941834

**Published:** 2022-10-03

**Authors:** Amani A. Mahbub, Akhmed Aslam, Mohamed E. Elzubier, Mohamed El-Boshy, Abdelghany H. Abdelghany, Jawwad Ahmad, Shakir Idris, Riyad Almaimani, Aiman Alsaegh, Mahmoud Zaki El-Readi, Mohammed A. Baghdadi, Bassem Refaat

**Affiliations:** ^1^ Laboratory Medicine Department, Faculty of Applied Medical Sciences, Umm Al-Qura University, Makkah, Saudi Arabia; ^2^ Department of Biochemistry, Faculty of Medicine, Umm Al-Qura University, Makkah, Saudi Arabia; ^3^ Biochemistry Department, Faculty of Medicine and Surgery, National University, Khartoum, Sudan; ^4^ Clinical Pathology Department, Faculty of Veterinary Medicine, Mansoura University, Mansoura, Egypt; ^5^ Department of Anatomy, Faculty of Medicine, Alexandria University, Alexandria, Egypt; ^6^ Biochemistry Department, Faculty of Pharmacy, Al-Azhar University, Assuit, Egypt; ^7^ Research Centre, King Faisal Specialist Hospital & Research Centre, Jeddah, Saudi Arabia

**Keywords:** testosterone, androgen receptor, oestrogen receptor, progesterone receptor, cell cycle, apoptosis

## Abstract

Although ovarian sex steroids could have protective roles against colorectal cancer (CRC) in women, little is currently known about their potential anti-tumorigenic effects in men. Hence, this study measured the therapeutic effects of 17β-oestradiol (E2) and/or progesterone (P4) against azoxymethane-induced CRC in male mice that were divided into (n = 10 mice/group): negative (NC) and positive (PC) controls, E2 (580 µg/Kg/day; five times/week) and P4 (2.9 mg/Kg/day; five times/week) monotherapies, and concurrent (EP) and sequential (E/P) co-therapy groups. Both hormones were injected intraperitoneally to the designated groups for four consecutive weeks. Similar treatment protocols with E2 (10 nM) and/or P4 (20 nM) were also used in the SW480 and SW620 human male CRC cell lines. The PC group showed abundant colonic tumours alongside increased colonic tissue testosterone levels and androgen (AR) and oestrogen (ERα) receptors, whereas E2 and P4 levels with ERβ and progesterone receptor (PGR) decreased significantly compared with the NC group. E2 and P4 monotherapies equally increased ERβ/PGR with p21/Cytochrome-C/Caspase-3, reduced testosterone levels, inhibited ERα/AR and CCND1/survivin and promoted apoptosis relative to the PC group. Both co-therapy protocols also revealed better anti-cancer effects with enhanced modulation of colonic sex steroid hormones and their receptors, with E/P the most prominent protocol. *In vitro*, E/P regimen showed the highest increases in the numbers of SW480 (2.1-fold) and SW620 (3.5-fold) cells in Sub-G1 phase of cell cycle. The E/P co-therapy also disclosed the lowest percentages of viable SW480 cells (2.8-fold), whilst both co-therapy protocols equally showed the greatest SW620 apoptotic cell numbers (5.2-fold) relative to untreated cells. Moreover, both co-therapy regimens revealed maximal inhibitions of cell cycle inducers, cell survival markers, and AR/ERα alongside the highest expression of cell cycle suppressors, pro-apoptotic molecules, and ERβ/PGR in both cell lines. In conclusion, CRC was associated with abnormal levels of colonic sex steroid hormones alongside aberrant protein expression of their receptors. While the anti-cancer effects of E2 and P4 monotherapies were equal, their combination protocols showed boosted tumoricidal actions against CRC in males, possibly by promoting ERβ and PGR-mediated androgen deprivation together with inhibition of ERα-regulated oncogenic pathways.

## 1 Introduction

Colorectal cancer (CRC) is the third most prevalent malignancy and ranked fourth amongst all cancer-related deaths globally (1, 2). Colon carcinogenesis is associated with aberrant increases in cyclin D1 (CCND1), CCND3, B-cell lymphoma 2 (BCL2), and survivin proteins, which induce cell cycle progression and inhibit cell death (3, 4). In the same vein, colon tumorigenesis also involves downregulations in the cell cycle inhibitors, cyclin-dependent kinase (CDK)-inhibitors (p21 and p27), alongside the pro-apoptotic molecules, cytochrome C (Cyto-C) and caspase-3 (Casp-3) (5–7). Although 5-Fluororacil is the chief chemotherapeutic drug used for the treatment of CRC, it has limited efficacy during the advanced stages of malignancy (8, 9).

Numerous epidemiological studies have shown that women of reproductive age have lower incidence of CRC compared to their counterpart age-matched men (10, 11). Furthermore, menopausal women using oestrogen-alone or combined with progesterone (P4) hormone replacement therapy (HRT) had significantly lower rates of CRC relative to age-matched nonuser women (10, 11). Additionally, normal colonic epithelium can generate and respond to sex steroid hormones, since enterocytes express the synthesising enzymes of 17β-oestradiol (E2), P4, and testosterone alongside their nuclear receptors (ERα, ERβ, PGR and AR) (12–15). Serum concentrations of E2 and/or P4 also correlated with significantly better prognosis in CRC patients, independent of their sex (14, 16–19). *In vitro* and *in vivo* experimental studies have likewise demonstrated suppression of cell proliferation and survival following treatment with E2 and P4 by ERβ and/or PGR-mediated anti-cancer activities (18–25). In contrast, circulatory testosterone levels and the expression of AR in neoplastic colonic tissues showed positive associations with tumour size, advanced clinical stage, and lower survival rates in both genders (15, 26–28). Therefore, it has been proposed that E2 and P could promote tumour suppressive effects, whilst testosterone may enhance CRC progression (29, 30).

To the best of our knowledge, only a single study measured the effects of E2 and/or P4 single and dual treatments against CRC in ovariectomised female rats, with the dual protocol showing better anti-cancer actions (31). However, the hormones in the dual therapy protocol were administrated concomitantly (31), which does not simulate the physiological hormonal chronological sequence in women of childbearing age (32). Additionally, no reports explored the anti-tumorigenic effects of E2 and P4 combined treatment in males. Hence, this study was design to measure the chemopreventive effects of exogenous female sex steroid hormones alone, as well as their concomitant and sequential dual therapy protocols against CRC in male mice. Moreover, the SW480 and SW620 human male colon cancer cell lines were treated with similar protocols to validate the findings of the animal studies.

## 2 Materials and methods

### 2.1 Chemicals and reagents

Azoxymethane (AOM; #A5486-100MG), 17β-oestradiol (E2; #E8875-5G) and progesterone (P4; #P0130-25G) were from Sigma-Aldrich Co. (St. Louis, MO, USA). Pan-species ELISA kits against E2 (#CEA461Ge), P4 (#CEA459Ge), and testosterone (#CEA458Ge), along with mouse-specific kits against survivin (#SEC045Mu), and cytochrome C (#SEA594Mu), were from Cloud-Clone Corp. (Houston, TX, USA). Furthermore, DMEM media (#10566032), foetal bovine serum (FBS; #A3160802), antibiotic-antimycotic solution (#15240062) and all the cell culture materials were from Thermo Fisher scientific (Waltham, MA, USA).

### 2.2 Experimental animal studies and treatment protocols

Sixty male BALB/c mice of 10 weeks of age and body weight ranging between 25-30 gm each were distributed into six equal groups (10 mice/group), as follows: the negative (NC) and positive (PC) controls, E2 and P4 monotherapy groups, the dual therapy groups that received E2 with P4 either concurrently (EP) or sequentially (E/P). All groups, except the NC animals, were injected with AOM for two successive weeks (10 mg/kg/week) to induce CRC, as reported earlier (33, 34). The mice were then housed in controlled environment (24°C ± 1 temperature and 12h light/dark cycle) and received standard laboratory diet with water *ad libitum* for 20 weeks. AOM is a carcinogen that is commonly used for the induction of CRC in murine mammals. This model emulates the clinical stages of CRC in humans by initially stimulating numerous pro-oncogenic pathways that subsequently promote the development of pre-neoplastic lesions, including mucin depleted foci (MDF), which later progress to invasive carcinoma after 15 weeks from the last injection of AOM (34, 35).

At week-21 post-AOM, E2 (580 µg/Kg/day; five times/week) and P4 (2.9 mg/Kg/day; five times/week) were freshly prepared in olive oil and injected intraperitoneally to the designated groups for four consecutive weeks. Moreover, the NC and PC groups received olive oil (200 µl) intraperitoneal injections as vehicle for four weeks. As per the dose conversion formula between human and mice (36), the injected amounts of hormones were equal to the highest daily doses of E2 (2 mg/day; 33.3 µg/kg/day) and P4 (10 mg/day; 166.7 µg/kg/day) recommended for postmenopausal women of 60 kg body weight (32). While the EP group were treated with both hormones concurrently for four weeks, the E/P group received E2 alone for two weeks followed by E2 and P combined therapy for another two weeks to mimic normal female reproductive endocrinology at childbearing age. The study was approved by the Committee for the Care and Use of Laboratory Animals at Umm Al-Qura University (AMSEC 22/05-10-20) and the experiments were concordant with the European guidelines for the care and use of laboratory animals.

#### 2.2.1 Collection of colonic specimens and enumeration of tumours

The mice were euthanised on the first day of week-25 post-AOM by cervical dislocation under anaesthesia as previously described (33). The colon from each mouse was harvested, infused with cold phosphate buffer saline (PBS), cut longitudinally, and preserved overnight between layers of filter papers saturated with 10% formalin (37). On the proceeding day, two researchers counted the numbers of tumours/colon by naked eye followed by cutting each colon into three equal segments corresponding to proximal, middle, and distal parts to caecum (37). Each segment was stained with 1% Alcian blue solution (#sc-214517; Santa-Cruz Biotechnology Inc.; CA, USA) in 3% acetic acid (pH 2.5) for 10 minutes. The numbers of MDF and small tumours that were not identified by gross examination were counted with a dissecting microscope (Human Diagnostics; Wiesbaden, Germany), as described earlier (37).

Each colonic segment was then cut longitudinally, and a piece was fixed in 10% formalin for 24h and processed by an automate Leica APS300 processor (Leica Microsystems; Wetzlar, Germany) prior to paraffin embedding. The residual fresh colonic tissues were homogenised in RIPA lysis buffer (#89900) with protease inhibitors (#78429; Thermo Fisher Scientific) for total protein extraction. The concentrations of extracted total protein were measured by a Pierce™ Rapid Gold BCA Protein Kit (#A53225; Thermo Fisher Scientific) and each sample was then diluted with deionized water (2000 µg/ml) to be used for ELISA.

#### 2.2.2 Colonic tumours histopathological features

Two skilled researchers examined all colonic tissue sections by a Leica DMi8 microscope (Leica Microsystems) following H&E staining. The histological features of adenocarcinomas in five random fields/section were reported by both researchers using a set of well-established criteria (37). An independent expert histopathologist re-evaluated the sections when both examiners widely disagreed about the histopathological characteristics of tumours.

#### 2.2.3 Immunohistochemistry

CCND1 (#55506) and CDK inhibitor-1A (p21; #37543) were detected by rabbit monoclonal antibodies (Cell Signaling Technology Inc.; Danvers, MA, USA). Moreover, rabbit polyclonal antibodies were used to detect AR (#PA5-85072) and ERβ (#PA1-310B), whereas PGR (#MA1-411) and ERα (#MA1-80216) were mouse monoclonal IgG antibodies (Thermo Fisher scientific). Five-μm sections were treated with a BLOXALL^®^ Solution (#SP-6000-100; Vector Laboratories Inc., CA, USA) for 15 min to block endogenous peroxidases. The sections were incubated overnight with the primary antibodies (1:200 concentration for all) at 4°C. After washing, the sections were treated with ImmPRESS^®^ HRP Horse Anti-mouse (#MP-7402) or anti-rabbit (#MP-7401) IgG Plus Polymer Peroxidase Kits, as per the manufacturer’s protocol (Vector Laboratories Inc.). Archived rat ovarian tissues were used as positive controls. The same protocol was also used with the negative control sections, but primary isotype mouse (#sc-2025) and rabbit (#sc-2027) IgG antibodies (Santa-Cruz Biotechnology) were used to control for non-specific staining. The sections were studied on a Leica DMi8 microscope and images were acquired from 10 random fields/section with a 20× objective. The IHC Image Analysis Toolbox in ImageJ software (https://imagej.nih.gov/ij/) was used to measure the protein expression, as described elsewhere (38, 39).

In brief, the IHC tool was used to precisely pinpoint the stained areas in each red/green/blue (RGB) image and were named as the region of interest (ROI). Subsequently, the numbers of stained pixels and their percentages (%ROI) in relation to the total numbers of pixels in each image were calculated by the software (40). Moreover, non-stained areas in RGB images (white colour) always generate the highest scores (255) by digital image analysis, whereas stained areas always have scores< 255. Hence, the IHC scores were calculated by the following equation, as previously described (40):


IHC stain intensity =(White unstained area score [255]− ROI stain score)× % ROI [ROI pixels /total image pixels × 100]

#### 2.2.4 Cell death by terminal deoxynucleotidyl transferase-dUTP nick end labelling assay

Cell apoptosis was detected in colonic tissues with a Click-iT™ Plus TUNEL Assay (#C10617; Thermo Fisher Scientific) and by following the manufacturer’s instructions. The co-detection of apoptotic bodies with cleaved Casp-3 was achieved by a sequential staining protocol, as described earlier (41, 42). Briefly, anti-cleaved Casp-3 rabbit IgG monoclonal antibodies (#9661; Cell Signaling Technology Inc.) were added (1:400 concentration) for 3h following completion of the TUNEL protocol. Next, the slides were incubated with donkey anti-rabbit IgG antibodies tagged with Alexa Fluor™ 555 (#A-31572; Thermo Fisher Scientific) for 30 min and DAPI (#D3571; Thermo Fisher Scientific) was used for counterstaining. A permanent fluorescence anti-fade mounting medium (#S3023; Dako, CA, USA) was used for cover-slipping and the slides were examined on a Leica DMi8 microscope with a 40× objective. Images were captured from 15 non-overlapping fields/section, and the numbers of apoptotic cells and stain intensity of cleaved Casp-3 were measured by ImageJ software, as previously reported (38, 43).

#### 2.2.5 Elisa

Colonic tissue homogenate samples were processed in duplicate on an automated ELISA machine (Human Diagnostics) to measure the levels of E2, progesterone and testosterone hormones alongside the concentrations of survivin and cytochrome C proteins.

### 2.3 *In vitro* experiments

Human SW480 and SW620 male colon cancer cell lines were obtained from the American Type Culture Collection (ATCC; MA, USA), cultured in DMEM containing 10% FBS and 1% antibiotic-antimycotic solution, and grown in a humified incubator at 37°C and 5% CO_2_. The concentrations (IC50) of E2 (10 nM) and/or P4 (20 nM) were established by the 3-(4,5-Dimethylthiazol-2-yl)-2,5-Diphenyltetrazolium Bromide (MTT) cytotoxicity assay at 72h as previously reported (data not shown).

Prior to flow cytometry analysis, the SW480 (2x10^5^) and SW620 (3x10^5^) cells were seeded in 6-well plates for 24h and then treated with E2 and P4 alone or combined (concomitant) for 48h. In addition, the sequential treatment involved the addition of E2 for 24h followed by P4 for another 24h, resulting in the following groups: untreated control (CT), E2 and P4 single therapies, and concomitant (EP) and sequential (E/P) co-therapies. The 48h time-point was used to ensure that any effects of dual treatments could be accurately analysed by cell cycle, apoptosis, and protein expression techniques.

#### 2.3.1 Cell cycle analysis

Following the different treatment regimens in the SW480 and SW620 cells, cell cycle analysis was performed with a NovoCyte 3000 flow cytometer (Agilent Technologies, CA, USA), as reported earlier (33). In summary, cells were trypsinised, washed with PBS (500× *g* for 5 min) and fixed in ice-cold 70% ethanol for 24h at 4°C. After PBS washing (600× *g*; 5 min/each), the cells were treated for 15 min with RNase A (20 µg/ml; #12091021; Thermo Fisher) and 2 µg/ml propidium iodide (PI; #P1304MP; Thermo Fisher). The cell numbers in the cell cycle phases (Sub-G1, G0/G1, S, G2/M) were then determined by the NovoExpress software cell cycle algorithm for 20,000 events (n = 3; data represented mean ± SD).

#### 2.3.2 Apoptosis assay

Cell apoptosis was measured by an Annexin V-FITC/PI Apoptosis Assay Kit (#V13245; Thermo Fisher Scientific) as per the kit’s protocol. Following the different treatments, the SW480 and SW620 cells were collected, washed twice with cold-ice PBS, and re-suspended in 100 µl of 1× Annexin V (AV) binding buffer. A mixture of AV-FITC (5 µl) and PI (1 µl) was then added to each of the SW480 and SW620 cell suspensions followed by incubation in the dark for 15 min at room temperature for cell staining. Next, 400 µl of the AV binding buffer were added, and the cells were placed on ice and instantly analyzed with the NovoCyte 3000 flow cytometry. The experiments were conducted in triplicate and the data show the percentage (mean ± SD) of cells in the different apoptosis stages as follows: live (unstained), early (AV+/PI-) and late apoptotic (AV+/PI+), and dead (AV-/PI+) cells.

#### 2.3.3 Western blot

Primary rabbit monoclonal antibodies from Cell Signaling Technology Inc. were used to detect CCND1, p21, CCND3 (#2936), p27 (#3686), BCL2 (#15071), Cyto-C (#4272), and cleaved Casp-3 by Western blot. The same primary antibodies used for the detection of AR, ERα ERβ and PGR by IHC were also utilised for Western blotting. Normalisation was done by GAPDH loading control mouse monoclonal antibodies (#MA5-15738-1MG; Thermo Fisher Scientific).

Following extraction of total proteins from each cell pellet, 50 µg/sample were loaded on gradient 4–20% Mini-PROTEAN^®^ TGX Stain-Free™ SDS-PAGE gels (#4568096; Bio-Rad Laboratories Inc.; CA, USA). A Trans-Blot^®^ Turbo™ Transfer System (Bio-Rad Laboratories Inc) was then used to transfer the protein samples onto 0.2 µm Trans-Blot^®^ Turbo™ PVDF membranes, followed by blocking with SuperBlock™ T20 buffer (TBS-T; #37543; Thermo Fisher Scientific) for 15 min. Subsequently, the membranes were incubated overnight at 4°C with the primary antibodies (1:1000 for all antibodies). In the next morning, the membranes were washed with TBS-T followed by incubation for 1h with WestVision™ (Vector Laboratories Inc.) peroxidase micropolymer-conjugated secondary anti-mouse (#WB-2000-.8) or anti-rabbit (#WB-1000-.8) IgG antibodies (1:10,000). Following washing, SignalFire^™^ Plus ECL Reagent (#12630; Cell Signaling Technology Inc.) was used for signal development. The images were captured by a ChemiDocTM XRS+ (Bio-Rad Laboratories Inc.) and band densitometry for each targeted protein was quantified and normalised against the corresponding GAPDH band by ImageJ software as reported earlier (44). Data are presented as mean ± SD of three blots/cell line for each protein of interest.

### 2.4 Statistical analysis

SPSS statistical analysis software version 25 was used for data analysis the data. Normality and homogeneity of all variables were assessed by the Kolmogorov and Smirnov’s test and the Levene test, respectively. One-way analysis of variance (ANOVA) with Tukey’s HSD or Games-Howell *post-hoc* tests were used for comparing between the different groups according to variance equality. Correlation studies were conducted by Pearson’s correlation test. P value< 0.05 indicated statistical significance.

## 3 Results

### 3.1 Treatment with ovarian sex steroid hormones in male mice and characteristics of CRC

**Figure 1:**
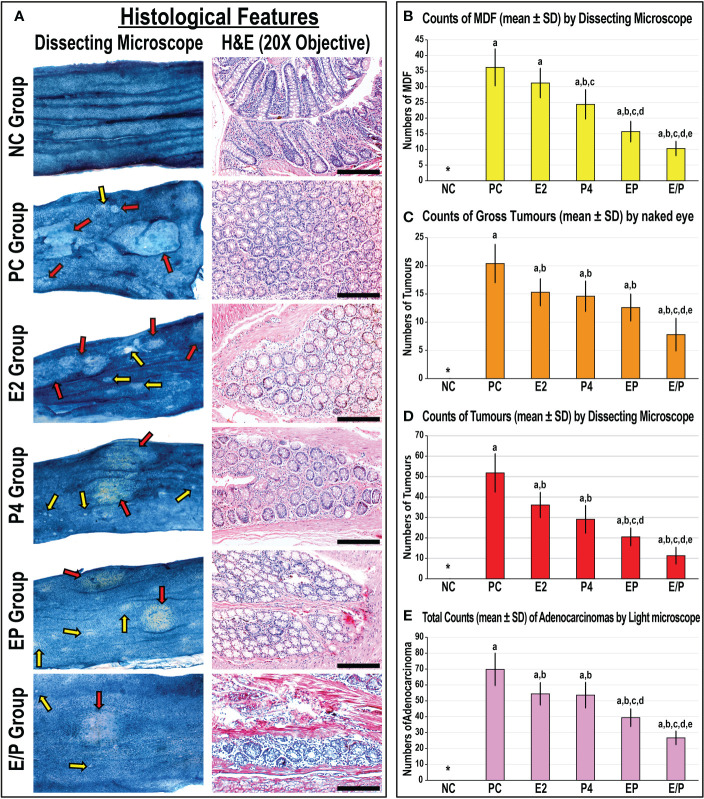
**(A)** Male mouse colon mucosa from all the study groups by dissecting microscope (×20 magnification; red arrow = tumours & yellow arrow = mucin depleted foci [MDF]) alongside colonic tissue sections from all groups by H&E stain (×200 magnification; scale bar = 15 μm). Furthermore, the numbers of **(B)** MDF, **(C)** gross tumours, **(D)** microscopic tumours, and **(E)** adenocarcinomas are shown as graph bars (mean ± SD; *Not detected; ^a^P< 0.05 compared with the NC group; ^b^P< 0.05 compared with the PC group; ^c^P< 0.05 compared with E2 monotherapy; ^d^P< 0.05 compared with P4 monotherapy and ^e^P< 0.05 compared with EP concurrent dual therapy protocol).

None of the animals died during the study period. Following dissection, the NC colonic specimens showed normal architecture and histology by dissecting and bright field microscopy, respectively (**Figure 1A**). In contrast, the numbers of MDF and gross tumours, as well as those of tumours detected by dissecting microscope, were abundant in the PC colonic tissues (**Figures 1A–D**). Moreover, numerous large adenocarcinomas with poorly to moderately differentiated histology were observed in the PC group colonic specimens by light microscope (**Figures 1A, E**).

While the MDF counts were markedly lower in the P4 group compared with the PC and E2 groups (**Figure 1B**), both monotherapies equally revealed significantly lower numbers of gross (**Figure 1C**) and microscopic (**Figure 1D**) tumours and adenocarcinomas (**Figure 1E**) relative to the PC group. Although both co-therapy protocols showed further reductions in the numbers of MDF, microscopic tumours, and adenocarcinomas compared with the PC, E2, and P4 groups, the numbers of gross tumours were comparable among the E2, P4 and EP groups (**Figure 1**). On the other hand, the sequential co-treatment (E/P) group showed the lowest numbers of MDF, gross tumours, and adenocarcinomas in comparison with the PC, both monotherapies, and the concomitant treatment (EP) groups (**Figure 1**).

### 3.2 Effects of ovarian sex steroid hormones single and combined therapies on cell cycle

#### 3.2.1 Markers of cell cycle in male mice colonic tissues

**Figure 2:**
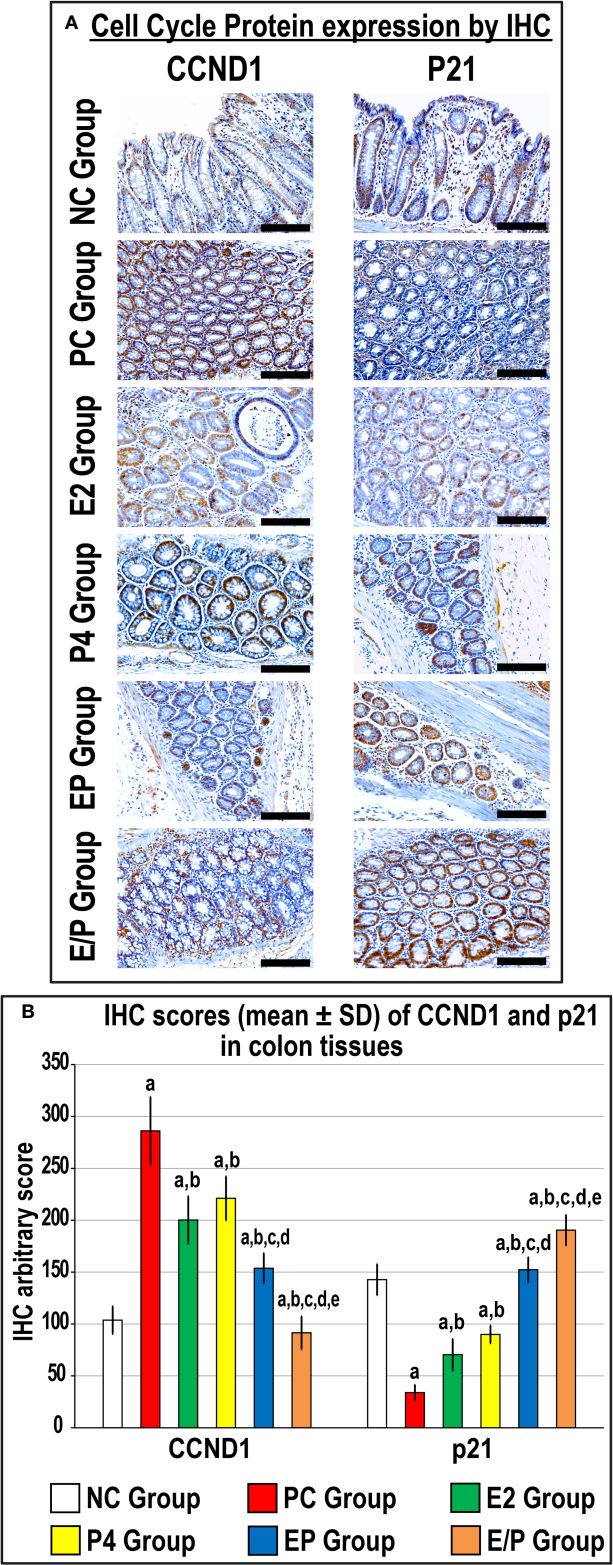
**(A)** Localisation of CCND1 and p21 proteins by immunohistochemistry (IHC) in male mouse colonic tissues from the different groups (20× objective; Scale bar = 15 μm). Moreover, **(B)** IHC arbitrary scores from the different groups are shown as graph bars (mean ± SD; ^a^P< 0.05 compared with the NC group; ^b^P< 0.05 compared with the PC group; ^c^P< 0.05 compared with E2 monotherapy; ^d^P< 0.05 compared with P4 monotherapy and ^e^P< 0.05 compared with EP concurrent dual therapy protocol).

The colonic specimens from the PC group revealed a marked increase in CCND1 and a substantial decrease in p21 proteins relative to the NC group (**Figure 2**). Both E2 and P4 single treatments showed equal significant reductions in CCND1 and marked elevations in p21 proteins compared with the PC group. While both dual therapy protocols revealed additional marked declines in CCND1 with concurrent augmentations in p21 proteins relative to the PC, E2, and P4 groups, the effects were significantly more pronounced in the sequential than the concomitant co-therapy approach (**Figure 2**).

#### 3.2.2 *In vitro* cell cycle arrest and expression of cell cycle regulatory molecules

**Figure 3:**
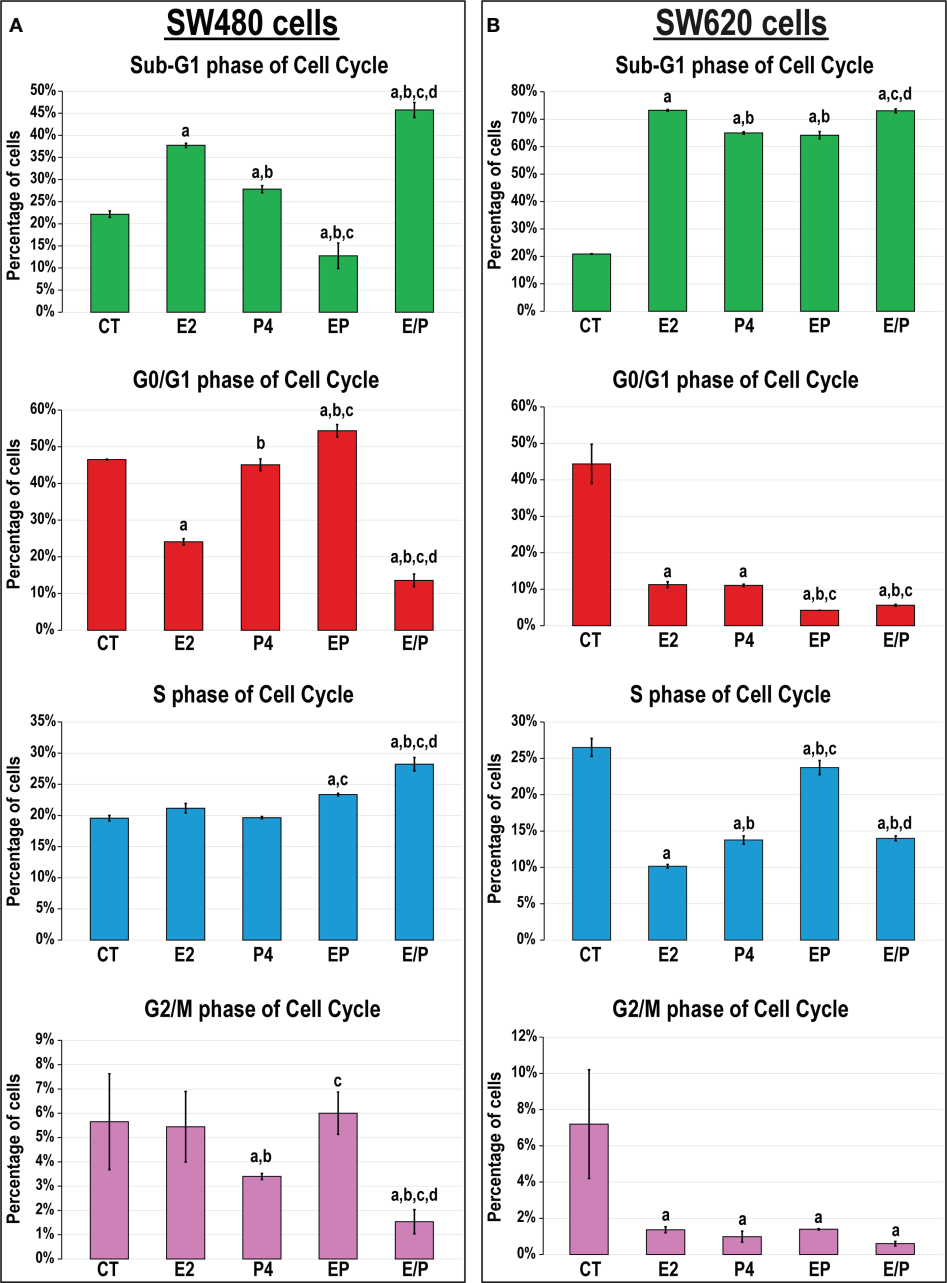
Percentages of cells (mean ± SD) in the different phases of cell cycle following the different treatment protocols with E2 and/or P4 for 48 hours in the **(A)** SW480 and **(B)** SW620 male colon cancer cell lines (mean ± SD; ^a^P< 0.05 compared with the CT group; ^b^P< 0.05 compared with the E2 monotherapy group; ^c^P< 0.05 compared with P4 monotherapy and ^d^P< 0.05 compared with EP concurrent dual therapy protocol).

The percentages of SW480 cells increased markedly with E2 (1.7-fold) and P4 (1.3-fold) single therapies, as well as with their sequential combination (E/P; 2.1-fold) in the sub-G1 phase, whereas their concurrent addition significantly reduced the cell numbers (EP; 1.7-fold), relative to untreated cells (**Figure 3A**). Moreover, the concomitant dual therapy revealed significant increases in the numbers of SW480 cells in the G0/G1-phase (1.2-fold), whereas the sequential approach demonstrated the highest percentages in S-phase of cell cycle (1.4-fold), compared with non-treated cells (**Figure 3A**). On the other hand, all treatments showed substantial increases in the percentages of SW620 metastatic cells in the sub-G1 phase compared with non-treated cells, with the E2 and E/P groups equally showing the highest numbers (3.5-fold for both; **Figure 3B**). Additionally, all single and dual therapies showed significant decreases in the cell numbers in all phases of cell cycle relative to untreated SW620 cells (**Figure 3B**).

**Figure 4:**
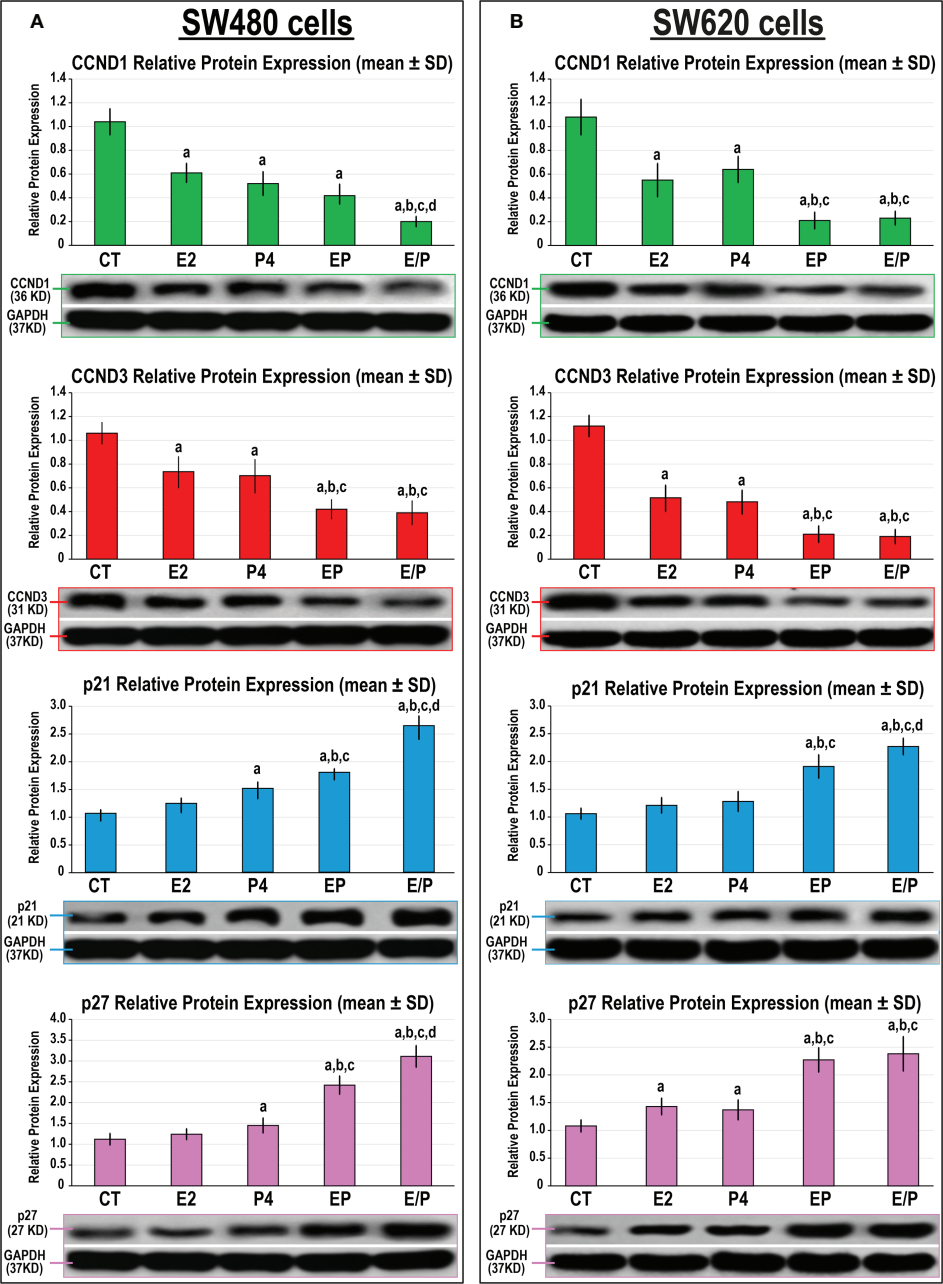
Detection of CCND1, CCND3, p21 and p27 proteins by Western blot alongside their relative protein expression (mean ± SD) following the different treatment protocols with E2 and/or P4 for 48 hours in the **(A)** SW480 and **(B)** SW620 male colon cancer cell lines (mean ± SD; ^a^P< 0.05 compared with the CT group; ^b^P< 0.05 compared with the E2 monotherapy group; ^c^P< 0.05 compared with P4 monotherapy and ^d^P< 0.05 compared with EP concurrent dual therapy protocol).

Both E2 and P4 monotherapies revealed similar significant declines in the expression of CCND1 and CCND3 proteins compared with untreated SW480 and SW620 cells (**Figure 4**). However, only P4 single therapy disclosed marked increases in the p21 and p27 proteins relative to non-treated and E2-treated SW480 cells (**Figure 4A**), whereas both proteins increased significantly and equally with E2 and P4 monotherapies in the SW620 cells compared with control cells (**Figure 4B**). Both dual-therapy protocols further reduced CCND1 and CCND3, whilst augmenting p21 and p27 proteins, compared with all monotherapies in both cell lines. Nevertheless, the lowest expression of CCND1 and CCND3, alongside the highest increases in p21 and p27 proteins, were detected with the sequential dual therapy approach in both cell lines relative to untreated cells, as well as the other treatment protocols (**Figure 4**).

### 3.3 Effects of ovarian sex steroid hormones on cell death and apoptosis markers

#### 3.3.1 *In vivo* colonic cell apoptosis and expression of apoptosis markers

**Figure 5:**
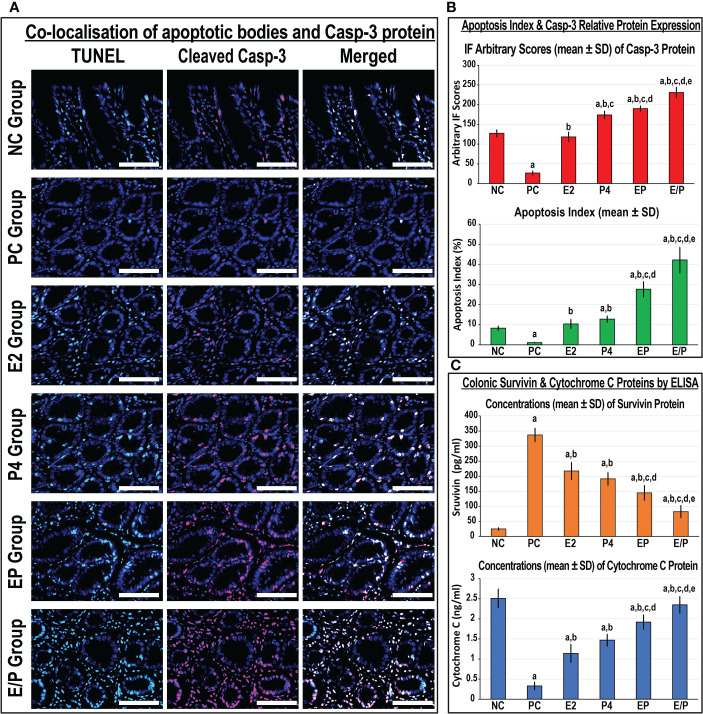
**(A)** Co-detection of apoptotic bodies by TUNEL (green) with cleaved Casp-3 (red) by immunofluorescence in male mouse colonic tissues from all the study groups (40× objective; scale bar = 8 µm). Moreover, **(B)** the relative expression of Casp-3 protein alongside apoptosis index and **(C)** in male mouse colonic tissues concentrations of survivin and cytochrome C proteins from all groups are as graph bars (mean ± SD; ^a^P< 0.05 compared with the NC group; ^b^P< 0.05 compared with the PC group; ^c^P< 0.05 compared with E2 monotherapy; ^d^P< 0.05 compared with P4 monotherapy and ^e^P< 0.05 compared with EP concurrent dual therapy protocol).

Apoptotic bodies and cleaved Casp-3 protein expression were mostly localised in glandular luminal and cryptic epithelial cells, as well as stromal cells in the NC colonic specimens (**Figure 5A**). The apoptosis index, cleaved Casp-3 protein expression, and colonic Cyto-C protein concentrations declined markedly and coincided with a substantial elevation in colonic tissue survivin protein levels in the PC group compared with the NC group (**Figure 5**). Both E2 and P4 monotherapies revealed significant declines in survivin and increases in Cyto-C colonic tissue concentrations in addition to higher apoptosis index and expression of Casp-3 protein relative to the PC group. Moreover, Casp-3 protein expression and apoptosis index were markedly higher in the P4 than the E2 group (**Figure 5B**), whereas the levels of survivin and Cyto-C proteins in colonic tissues were equal between both monotherapy groups (**Figure 5C**). Both dual therapy protocols showed significantly higher apoptosis index and Casp-3 protein expression, with markedly elevated Cyto-C and lower survivin protein concentrations in colonic tissues, compared with the PC, E2, and P4 groups (**Figure 5**). Nonetheless, the maximal increases in apoptosis index, cleaved Casp-3 expression, and cyto-C concentrations, with the lowest survivin levels, were detected in the sequential protocol compared with the PC, E2, P4, and EP groups (**Figure 5**).

#### 3.3.2 *In vitro* cell apoptosis and expression of apoptosis markers

**Figure 6:**
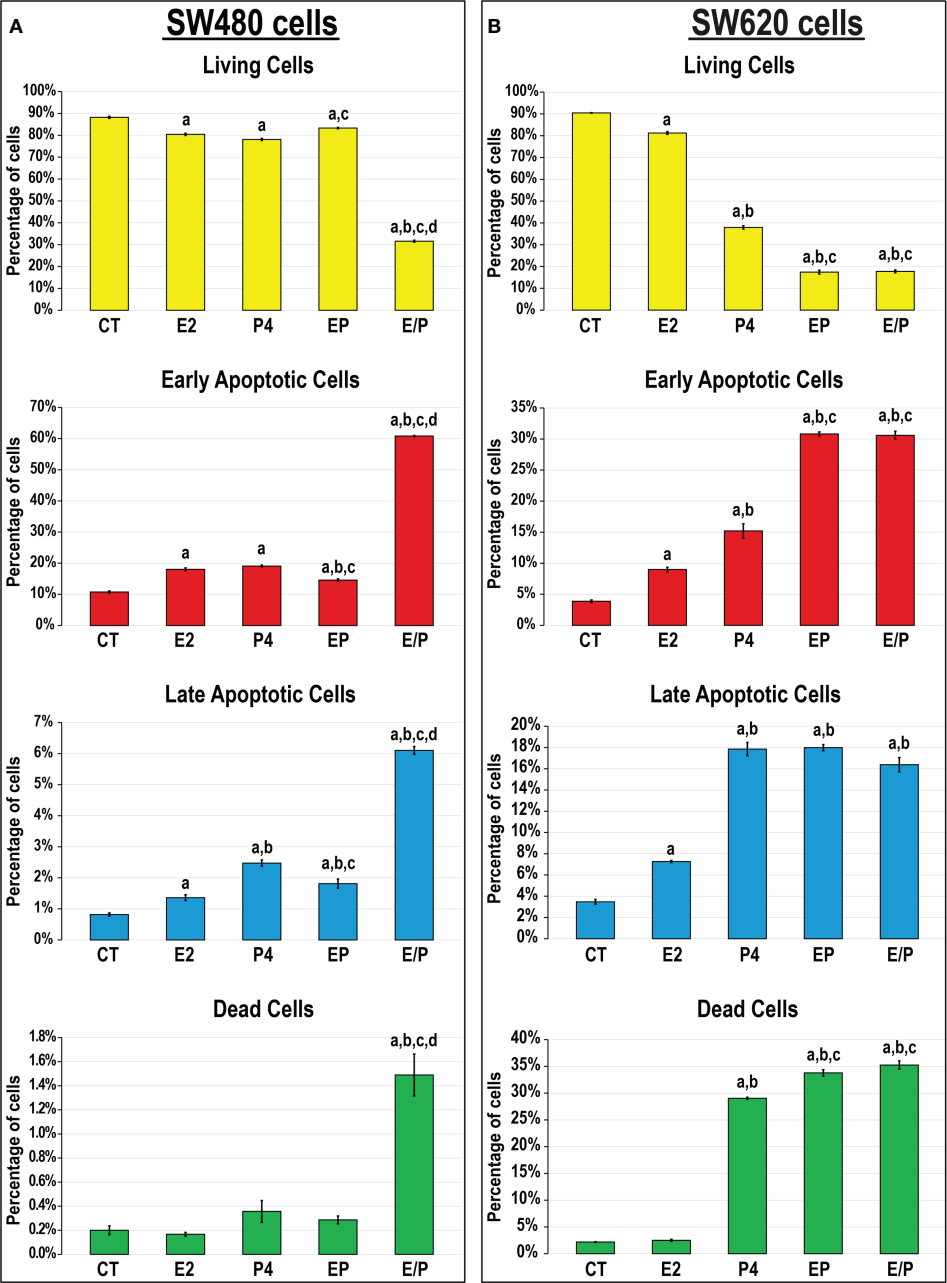
Percentages (mean ± SD) of living, early and late apoptotic alongside dead cells following the different treatment protocols with E2 and/or P4 for 48 hours in the **(A)** SW480 and **(B)** SW620 male colon cancer cell lines (mean ± SD; ^a^P< 0.05 compared with the CT group; ^b^P< 0.05 compared with the E2 monotherapy group; ^c^P< 0.05 compared with P4 monotherapy and ^d^P< 0.05 compared with EP concurrent dual therapy protocol).

While E2, P4 and EP treatments showed similar marginal, but significant, reductions in SW480 cell viability relative to control cells (1.1-fold for all), the E/P group displayed the highest frequency of apoptosis (2.8-fold) that was depicted by marked increases in the numbers of early (5.6-fold) and late (3.1-fold) apoptotic cells relative to all groups (**Figure 6A**). In the SW620 cells, E2 monotherapy showed a limited significant decline in cell viability (1.1-fold), whereas P4 disclosed substantially lower numbers of living cells (2.4-fold) with increased early (3.9-fold) and late (5.1-fold) apoptosis, compared with non-treated cells (**Figure 6B**). On the other hand, the lowest numbers of viable SW620 cells were equally detected in the EP and E/P groups (5.2-fold for both) and coincided with the highest increases in the percentages of early apoptotic cells (7.9-fold for both) compared with all groups. However, the numbers of late SW620 apoptotic cells were equal among the P4, EP, and E/P groups (**Figure 6B**).

**Figure 7:**
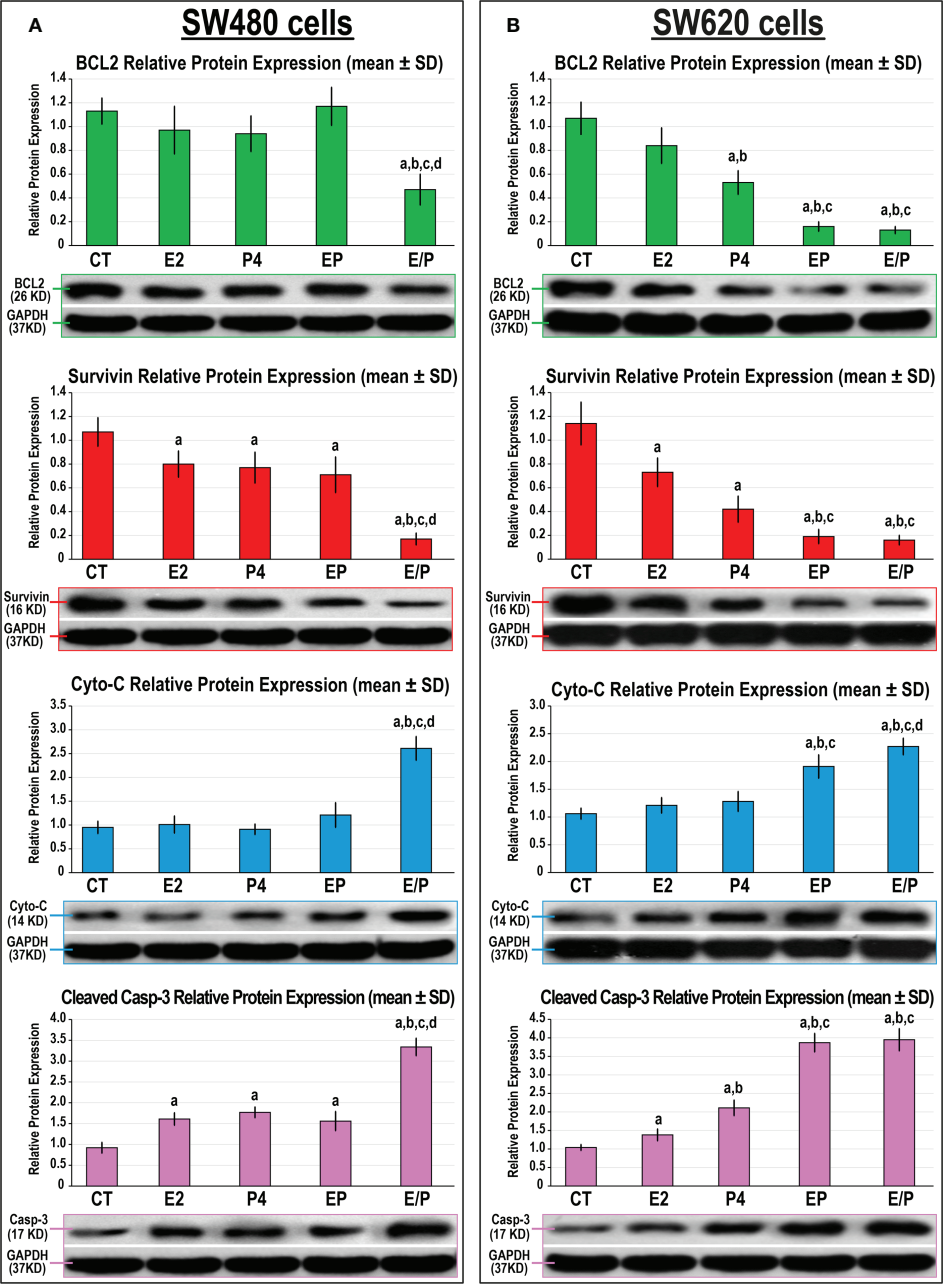
Detection of BCL2, survivin Cytochrome C and cleaved caspase-3 proteins by Western blot alongside their relative protein expression (mean ± SD) following the different treatment protocols with E2 and/or P4 for 48 hours in the **(A)** SW480 and **(B)** SW620 male colon cancer cell lines (mean ± SD; ^a^P< 0.05 compared with the CT group; ^b^P< 0.05 compared with the E2 monotherapy group; ^c^P< 0.05 compared with P4 monotherapy and ^d^P< 0.05 compared with EP concurrent dual therapy protocol).

Both monotherapies and their concomitant combination equally showed marked decreases in the protein expression of survivin with increases in Casp-3 protein, whereas they had no effect on BCL2 and Cyto-C proteins, compared with untreated SW480 cells (**Figure 7A**). In contrast, SW480 cells treated with the E/P sequential protocol revealed the maximal inhibitions in BCL2 and survivin proteins that concurred with the highest increases in Cyto-C and Casp-3 proteins compared with all groups. In the SW620 cells, single treatments with E2 and P4 were associated with marked decreases in survivin and increases in Casps-3 proteins relative to untreated cells (**Figure 7B**). Although the expression of BCL2 in the SW620 cells was comparable between the E2, P4, and control groups, the effects of P4 monotherapy on the expression of the remaining cell proliferation and cell apoptosis markers were significantly more prominent than E2 single therapy (**Figure 7B**). However, the concomitant and sequential co-therapy approaches in the SW620 cells equally exhibited the lowest BCL2 and survivin proteins with the maximal increases in Cyto-C and Casp-3 proteins compared with all other therapies (**Figure 7B**).

### 3.4 Effects of the different treatment protocols on colonic expression of sex steroid receptors

#### 3.4.1 Sex steroids hormones and their receptors in colonic tissues

**Figure 8:**
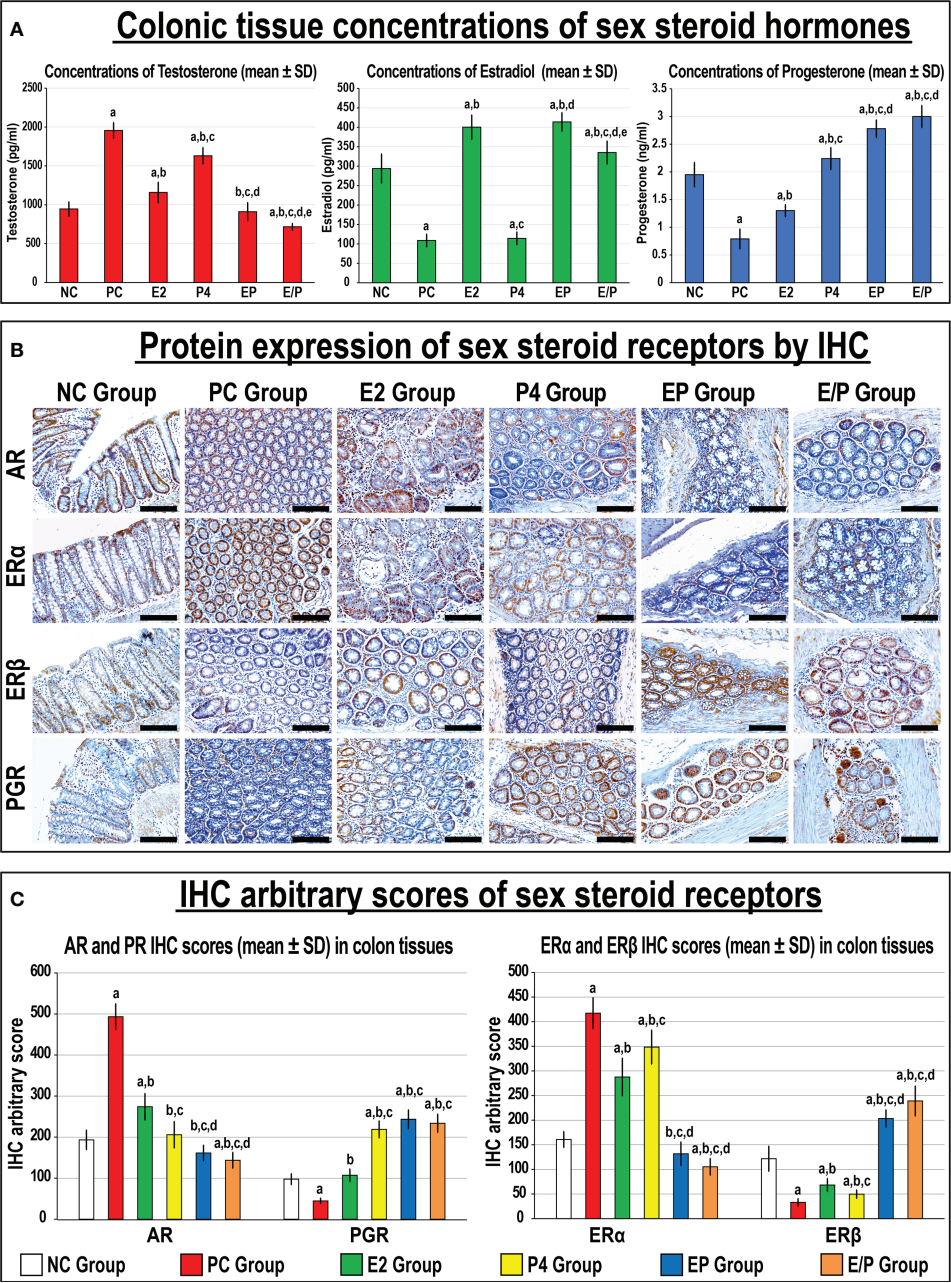
**(A)** Concentrations of testosterone, 17β-oestradiol, and progesterone hormones with **(B)** localisation of androgen (AR), progesterone (PGR) and oestrogen (ER) α and β receptors by immunohistochemistry (IHC) in male mouse colonic tissues from all the study groups (20× objective; Scale bar = 15 μm) and **(C)** the IHC arbitrary scores from the different groups are shown as graph bars (mean ± SD; ^a^P< 0.05 compared with the NC group; ^b^P< 0.05 compared with the PC group; ^c^P< 0.05 compared with E2 monotherapy; ^d^P< 0.05 compared with P4 monotherapy and ^e^P< 0.05 compared with EP concurrent dual therapy protocol).

Testosterone, 17β-oestradiol, and progesterone hormones were detected in colonic tissue homogenates from the NC group (**Figure 8A**). Additionally, the protein expression of AR, ERα, ERβ, and PGR were detected by IHC in the NC colonic tissues with exclusive nuclear localisation in glandular epithelial cells (**Figure 8B**). The PC colonic tissues showed significantly higher concentrations of testosterone with marked decreases in E2, and P4 levels compared with the NC group. Moreover, the protein expression of AR and ERα increased, whilst ERβ and PGR were lower, in the PC relative to the NC colonic specimens (**Figures 8B, C**). While testosterone significantly declined and E2 increased with all single and dual therapies, only mice treated with P4 alone or combined with E2 exhibited marked increases in colonic tissue progesterone levels, compared with the PC group (**Figure 8A**). Moreover, all treatment protocols were associated with significant decreases in the protein expression of AR and ERα, whilst ERβ and PGR increased, compared with the PC group (**Figures 8B, C**). Furthermore, the combined therapy protocols demonstrated more significant declines in colonic testosterone concentrations with AR and ERα protein expression, whereas colonic E2 and P4 levels with ERβ and PGR proteins were augmented, compared with both monotherapy groups. Although the protein expression of the targeted receptors was equal in the EP and E/P co-therapy groups, colonic testosterone and E2 levels were significantly lower in the latter group (**Figure 8**).

Additionally, colonic tissue concentrations of testosterone showed strong negative correlations with those of E2 (r = -0.818; P< 0.0001) and P4 (-0.690; P< 0.0001) levels. However, the colonic tissue E2 and P4 levels were weakly and positively linked together (r = 0.406; P< 0.0001). Colonic testosterone levels also displayed direct correlations with the numbers of MDF (r = 0.698), gross (r = 0.652) and microscopic (r = 0.774) tumours, and adenocarcinomas (r = 0.704; P< 0.0001 for all). In contrast, colonic E2 and P4 levels correlated negatively with the numbers of MDF (r = -0.355 & r = -0.624, respectively), gross (r = -0.346 & r = -0.449) and microscopic (r = -0.430 & r = -0.643) tumours, and adenocarcinomas (r = -0.401 & r = -0.497), respectively (P< 0.001 for all).

#### 3.4.2 *In vitro* protein expression of sex steroid receptors

**Figure 9:**
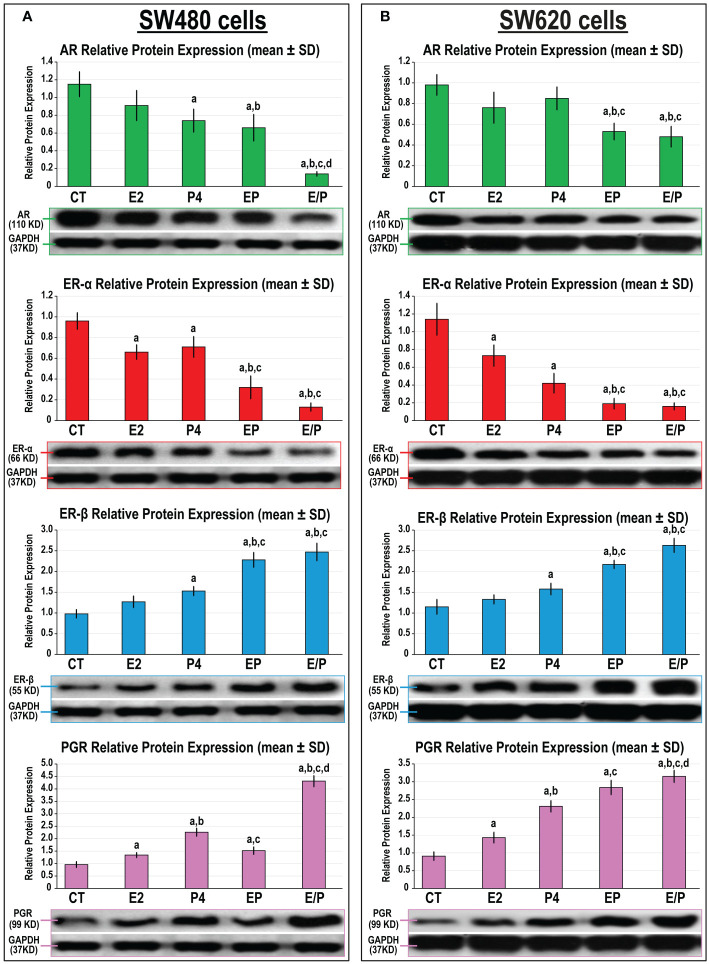
Detection of androgen (AR), progesterone (PGR) and oestrogen (ER) α and β receptors by Western blot alongside their relative protein expression (mean ± SD) following the different treatment protocols with E2 and/or P4 for 48 hours in the **(A)** SW480 and **(B)** SW620 male colon cancer cell lines (mean ± SD; ^a^P< 0.05 compared with the CT group; ^b^P< 0.05 compared with the E2 monotherapy group; ^c^P< 0.05 compared with P4 monotherapy and ^d^P< 0.05 compared with EP concurrent dual therapy protocol).

In agreement with the animal studies, the protein expression of AR, ERα, ERβ, and PGR was detected in the SW480 and SW620 male cell lines (**Figure 9**). While E2 single treatment only showed a significant decrease in ERα protein, P4 monotherapy was associated with marked declines in AR and ERα together with increases in ERβ and PGR proteins, relative to untreated SW480 cells (**Figure 9A**). Similar effects were also observed in the SW620 following E2 and P4 monotherapies (**Figure 9B**). The protein expression of AR and ERα further declined, whilst ERβ and PGR increased, with the EP and E/P co-therapy protocols compared with control, E2, and P4 groups in both cell lines. Although the SW480 and SW620 protein expression of ERα and ERβ was equivalent in both dual therapy groups, the E/P group showed the lowest AR expression that coincided with the maximal increase in PGR protein relative to the EP group in both cell lines (**Figure 9**).

## 4 Discussion

This study explored the effects of 17β-oestradiol (E2) and progesterone (P4) monotherapies, as well as their concomitant and sequential combinatory actions against AOM-induced CRC in male mice. Similar treatment regimens were also used in the SW480 primary and SW620 metastatic male colon cancer cell lines. AOM-induced CRC is a well-established pre-clinical model that mimics the different phases, as well as shares several molecular oncogenic pathways, of sporadic phenotype of human colon neoplasia (34, 35). Following injection, the metabolites of AOM accumulate in colonic cells and incite aberrant molecular and histological alterations that cause mucin depletion and formation of MDF, which are pre-malignant lesions frequently used as biomarkers for measuring the efficacy of chemopreventive agents in preclinical studies (34, 35).

Herein, the PC mice had abundant MDF with colonic neoplastic lesions that coincided with significantly higher intratumoral concentrations of total testosterone with AR and ERα protein expression, whereas E2 and P4 levels and the protein expression of ERβ and PGR decreased, relative to the NC group. The PC tumorous tissues also showed marked increases in CCND1 protein expression and higher levels of survivin protein, whilst p21, Casp-3 and Cyto-C proteins and apoptosis index declined, relative to the NC mice. Moreover, the colonic tissue concentrations of testosterone correlated positively, whereas E2 and P4 negatively, with the numbers of preneoplastic and neoplastic lesions.

In agreement with our results, the risk of CRC increased by > 20% following oophorectomy and the odds augmented substantially with bilateral rather than unilateral ovariectomy (45, 46). Moreover, higher serum levels of female reproductive steroid hormones alongside increased expression of ERβ and/or PGR in malignant tissues correlated with better prognosis in male and female CRC patients (14, 16–19). In contrast, elevated systemic testosterone levels and higher expression of ERα and AR proteins in CRC were associated with larger tumours, poor differentiation, advanced clinical stages, and worse outcomes in both genders (15, 26–28). Hence, our data and results from prior reports (14–16, 19, 28, 45, 46) suggest that testosterone with AR and ERα could promote CRC development, while E2 and P4, alongside ERβ and PGR, may act as tumour suppressors.

The actions of E2 in CRC are receptor-dependent, and several studies have shown that ERα is oncogenic, whilst ERβ exerts anti-tumorigenic actions (47). In essence, both ERα and ERβ are expressed by normal male and female colonic epithelium, and the latter is predominant (12–15). However, ERα increases, whilst ERβ declines significantly during colon carcinogenesis, and the former receptor is believed to transduce oncogenic activities (48, 49). E2 therapy induced cell cycle progression and promoted cell survival by ERα-mediated mechanisms, whereas its treatment also triggered apoptosis by activating Casp-3 *via* ERβ in DLD1 CRC cells (50). Others have also shown that ERα provoked colon tumorigenesis by stimulating the Wnt/β-catenin oncogenic pathway (51, 52). Moreover, ERα and ERβ revealed opposing effects on cell cycle progression in HeLa cells by upregulating and inhibiting CCND1 protein, respectively (53). ERβ overexpression in several human CRC cell lines also suppressed proliferation and induced apoptosis by increasing p21 and p27 cell cycle inhibitors, and Casp-3 and Cyto-C pro-apoptotic proteins, whilst reducing BCL2 and survivin anti-apoptotic proteins (18, 20–23). Similarly, prolonged use of natural and synthetic P4 was inversely correlated with the prevalence and recurrence of CRC in menopausal women (54). Lower PGR expression in colonic neoplastic tissues was also associated with poor prognosis and significantly lower survival rates in both genders (55). Concurrently, P4 treatment inhibited growth in human LoVo, HT29, HCT116, SW480 and SW620, as well as, murine MC38 colon cancer cells by decreasing several cell cycle inducer proteins and cell survival markers, whilst simultaneously increasing cell cycle suppressors and pro-apoptotic molecules (19, 24, 25). Moreover, *in vitro* treatment with folic acid inhibited proliferation in COLO-205, HT29, and LoVo CRC cells by PGR-mediated pathways (56).

In the present study, E2 and P4 single treatments equally reduced the counts of colonic tumours and adenocarcinomas compared with the PC animals. E2 and P4 monotherapies were also associated with significant declines in colonic CCND1, survivin, and BCL2 proteins alongside increases in p21, Cyto-C and Casp-3 proteins, and apoptosis index relative to the PC group. Additionally, E2 and P4 single therapies markedly increased the cell numbers in the Sub-G1 cell cycle phase, as well as, reduced the percentages of viable cells in the SW480 and SW620 cell lines compared with untreated cells. The protein expression of p21, p27, Cyto-C and Casp-3 also increased, whilst CCND1, CCND3, BCL2 and survivin proteins declined, following single treatments with E2 and P4 in the cell lines used. Furthermore, E2 and P4 monotherapies were associated with significant increases in ERβ and PGR alongside inhibitions in ERα both *in vivo* and *in vitro*. The present data correlate with many earlier studies and emphasise the chemopreventive effects of E2 that could involve inhibition of CRC progression by ERβ-induced cell cycle arrest and apoptosis in malignant enterocytes (18, 20–23, 57). Our study also underscores the previously reported anti-cancer activities of P4 against CRC (19, 24, 25, 56), which appear to be equal to those of E2 monotherapy, both *in vivo* and *in vitro*.

Indeed, the molecular pathways mediated *via* ERs and PGR interact together, and the activation of one receptor could influence the activities of the other in neoplastic diseases (58). In this context, the activation of ERβ (57) and PGR (59) halted the growth of breast cancer cells by inhibiting ERα-induced oncogenic actions. Notably, genetic studies have also shown that both PGR and ERβ pathways interacted together in CRC, and PGR-induced anti-tumorigenic effects were dependent on the activities of ERβ in malignant tissues (60). Furthermore, concomitant E2 and P4 dual therapy markedly increased the protein expression of ERβ, PGR, and Casp-3 alongside cell apoptosis, whereas downregulated the markers of cell proliferation, compared with single hormonal therapies in ovariectomised female rat model of CRC (31). However, none of the prior studies measured the potential anti-cancer effects of E2 and P4 sequential therapy in CRC, which imitates the normal sequence of reproductive endocrinology in women.

The current findings displayed boosted anti-cancer effects for E2 and P4 simultaneous and sequential co-therapy protocols that were manifested by marked reductions in tumour counts compared with the PC and both monotherapy groups. Likewise, both regimens of combined hormonal therapy disclosed significantly higher numbers of apoptotic SW480 and SW620 cells relative to non-treated and both monotherapy groups. However, the anti-cancer effects were substantially more pronounced with the sequential compared with the concomitant dual therapy protocol in mice and in the SW480 cells, whereas both protocols had equal apoptotic effects in the SW620 cells. Furthermore, the use of both hormones alone or combined revealed marked upregulations in ERβ and PGR alongside declines in ERα protein expression in neoplastic tissues. Concurrently, both co-therapy protocols showed significantly higher p21 and Casp-3 protein expression with colonic Cyto-C protein levels, whereas CCND1 and survivin proteins decreased markedly and coincided with a lower apoptosis index, compared with the PC, E2 and P4 groups. Collectively, our data and the earlier studies suggest that E2 and/or P4 could provide alternative therapeutic approaches against CRC and their efficacy could be dependent on the expression profiles of ERs and PGR in malignant colonic tissues (31, 57, 59, 60). Additionally, we hypothesise that the sequential hormonal therapy with E2 followed by P4 could be a superlative regimen against early stages of CRC, whereas their concomitant combination could be more appropriate for metastatic colon cancer. Proposed anti-cancer mechanisms for the combined protocols may involve enhanced expression of ERβ and PGR that subsequently inhibits ERα-mediated oncogenic effects with concurrent induction of cell cycle arrest and apoptosis in malignant enterocytes (31, 57, 59, 60).

Our results also revealed marked decreases in colonic tissue testosterone concentrations with E2 and/or P4 single and dual treatments. Moreover, both female sex hormones also decreased the expression of AR *in vivo* and *in vitro*. Hence, we speculate that the observed additive anti-tumorigenic effects for the combined hormonal therapy could also be related to the suppressive effects of E2 and P4 on colonic testosterone concentrations and expression of AR, which are believed to trigger tumorigenic activities (15, 27). Although there are no reports related to the effects of female sex steroid hormones on colonic AR expression, E2 and/or P4 therapies in transgender women markedly decreased serum levels of testosterone (61, 62). Additionally, both hormones decreased the expression of AR in a variety of tissues, including breast cancer and endometrium (63, 64). Therefore, we suggest that dual activation of ERβ and PGR by their ligands could initiate a cascade of cellular anti-cancer events involving inhibition of cancer progression induced *via* AR (15, 27) and ERα (57, 59) pathways alongside promoting anti-proliferative and pro-apoptotic activities (31, 60).

Nevertheless, the present study has several limitations. Firstly, we only measured the effects of the targeted female hormones in male mice and male human colon cancer cells, and future studies should investigate the anti-cancer hormonal actions in females, both *in vivo* and *in vitro*. Further studies are also required to explore the rapid non-genomic effects of E2 and P4 in colon cancer to fully elucidate the mechanisms underlying their anti-cancer effects in CRC (65, 66). Moreover, the chemopreventive effects of androgen deprivation treatment, with and without E2 and/or P4, against CRC, should also be studied to corroborate our observations.

In conclusion, CRC was associated with abnormal alterations in colonic levels of testosterone, 17β-oestradiol and progesterone and their nuclear receptors showed aberrant expression in malignant cells. Moreover, E2 and P4 monotherapies equally reduced the numbers of malignant lesions and colonic testosterone levels alongside AR and ERα protein expression, whilst upregulated ERβ and PGR and promoted cell cycle arrest and apoptosis in male mice, as well as in human male colon cancer cell lines. In contrast, the greatest anti-cancer activities were seen with the E2 and P4 combination protocols that could provide a superlative alternative therapeutic strategy against CRC, possibly by ERβ and PGR-mediated androgen deprivation and inhibition of ERα-induced cancer progression. However, more studies are needed to measure the effects of E2 and P4 single and dual therapies, with and without anti-androgen hormone therapy and/or chemotherapy (e.g., 5-FU), in male and female experimental models to define their precise therapeutic values in CRC.

## Data availability statement

The original contributions presented in the study are included in the article/supplementary material. Further inquiries can be directed to the corresponding author.

## Ethics statement

The animal study was reviewed and approved by The Committee for the Care and Use of Laboratory Animals at Umm Al-Qura University (AMSEC 22/05-10-20) and the experiments were concordant with the European guidelines for the care and use of laboratory animals.

## Author contributions

Conceptualization: AM, AKA and BR; Methodology: AKA, ME, ME-B, AHA, SI, JA, RA, AIA and MB; Investigation: AKA, AIA and BR; Visualization: BR and AHA; Validation: AKA, BR, and MZ-R; Formal analysis: BR and MZ-R; Data curation: AM, AKA and BR; Supervision: AM, AKA, ME-B., AHA, AIA and MZ-R; Funding acquisition: AM, AKA and BR; Resources: AM and BR; Project administration: AM and BR; Writing—original draft: AM and BR; Writing—review and editing: AKA. All authors have read and approved the manuscript.

## Funding

This work was supported by the Deanship of Scientific Research in Umm Al-Qura University [Grant Code: 19-MED-1-03-0007]. The funding organization was not involved in the design of the study; the collection, analysis, and interpretation of data; writing the report; and did not impose any restrictions regarding the publication of the report.

## Conflict of interest

The authors declare that the research was conducted in the absence of any commercial or financial relationships that could be construed as a potential conflict of interest.

## Publisher’s note

All claims expressed in this article are solely those of the authors and do not necessarily represent those of their affiliated organizations, or those of the publisher, the editors and the reviewers. Any product that may be evaluated in this article, or claim that may be made by its manufacturer, is not guaranteed or endorsed by the publisher.
